# Human detection of AI-generated faces and voices is not domain-general

**DOI:** 10.1038/s41598-026-59364-3

**Published:** 2026-06-22

**Authors:** Matthew Ivory, Carolyn McGettigan, Helen E. Nuttall, Sophie J. Nightingale

**Affiliations:** 1 Department of Psychology, Lancaster University, Fylde Building, Lancaster, LA14YW UK; 2 Department of Speech, Hearing and Phonetic Sciences, University College London, Chandler House, 2 Wakefield Street, London, WC1N 1PF UK

**Keywords:** Artificial intelligence, Human detection, Face, Voice, Deepfake, Neuroscience, Psychology, Psychology

## Abstract

Recent technological advances have resulted in synthetic faces and voices being perceptually indistinguishable from real faces and voices in typical populations. Faces and voices possess rich personal and social information, meaning synthetic faces and voices, commonly known as “deepfakes” can be used for identity theft, financial fraud, and misinformation campaigns. It is currently unknown whether detection of real versus synthetic content is modality-specific, or whether it generalizes across sensory domains. We conducted a preregistered study in which participants classified real and AI-generated faces and voices. Performance for both face and voice classification was significantly above chance. Using signal detection theory to analyze individuals’ ability to classify stimuli, we observed no evidence of a domain-general effect, indicating detection ability may not generalize across face and voice domains and is instead domain-specific. Participants’ confidence tracked accuracy for faces but not for voices, suggesting metacognitive insight may be modality-specific. The findings are discussed in terms of whether the absence of an effect reflects classification driven solely by domain-specific abilities or arises from the experimental design itself. Ultimately, in applied contexts, it is important to recognize that expertise in detecting synthetic content might be modality specific.

## Introduction

Generative Artificial Intelligence (AI) has democratized the ability to synthesize highly sophisticated audio, image, and video content—almost anyone can now generate content showing people doing and saying things that do not reflect reality. This synthetic content can be used for sinister and nefarious purposes, such as creating AI-generated non-consensual intimate imagery by superimposing an individual’s face into the scene^1^, to commit identity theft via synthetic images or speech^2^, or even impersonating politicians’ voices to spread misinformation during election campaigns^3^. Due to the relative ease of generating synthetic content and distributing it via social media or other channels, it is important to understand how humans perceive and distinguish between real and synthetic faces and voices. Both faces and voices are heavily loaded with personal and social context that determine how we trust associated information, and whilst research has been carried out with faces and voices independently, it is currently unknown as to whether individuals possess a modality-general ability to detect synthetic content or if detection abilities are modality specific. Accordingly, the current research seeks to address this gap by testing for the existence of a modality-general ability to identify real content from synthetic content. That is, do humans possess a “realness” detection ability, particularly for faces and voices?

In response to the use of synthetic content in scenarios designed to cause harm, disarray, and confusion, there have been technical solutions to identify such content^4^, such as tracking where video subjects look^5^ or analyzing audio files for artefacts (see Dagar et al.^6^ for a comprehensive review of technical detection techniques). Many of these techniques are asynchronous and cannot be used in real-time, as well as being inaccessible to the general public, meaning that people are still the first defense in distinguishing between real and synthetic content.

Artificially synthesized faces have been reported to be indistinguishable from real faces –perhaps due to their averageness— and perceived as more trustworthy^7,8^. Further, evidence suggests that synthetic and real faces are processed in a similar manner^9^, potentially leveraging the same face processing perceptual mechanisms. Average detection rates typically fall around chance^7,10,11^, and interventions such as reminders and advice do not always significantly improve detection^12^. Some evidence exists for improvements in performance when feedback is offered, but performance remains low^7^, with some training paradigms not improving classification rate per se, but increasing scepticism over authenticity^11^. This emphasizes the difficulty of distinguishing real faces from synthetic ones, a problem that may only be exacerbated as generative AI presents an ever-evolving landscape, with new or updated models released frequently.

A similar trend has been observed for voices, with real and synthetic voices being difficult to correctly classify—human performance is typically at, or just above, chance^13,14^. Research has also shown that exposure to feedback when classifying real and synthetic voices marginally boosts performance^15^. Differences have also been reported between types of synthetic voices. Text-to-speech voices (generated using predefined, generic vocal characteristics) are more easily detected than voice clones (synthetic speech replicating an individual’s unique vocal characteristics), though accuracy remains below 60%^14^; voice clones were often perceived as indistinguishable from real voices. This finding is not consistent, with non-English synthetic speech being reported as less realistic due to the pattern of speech^16^ suggesting that intonation and inflection are key determinants of realism judgements. Although research on human perception of synthetic voices is growing at pace, there is still relatively less work in this space compared with synthetic faces simply because the models for image generation were released earlier than those for voice generation.

Judgments of realism are not determined by perceptual evidence alone but are also shaped by systematic cognitive biases. According to Truth-Default Theory^17^, individuals typically assume information is authentic unless sufficient evidence triggers suspicion. This “truth-default” state implies a bias toward classifying stimuli as real, particularly in the absence of explicit cues or incentives to examine and question the authenticity. As a result, observed performance in synthetic content detection tasks likely reflects a combination of perceptual sensitivity and decisional bias, complicating the interpretation of accuracy measures and raising the possibility that apparent individual differences may partly reflect a shared tendency to evaluate information as real. Within synthetic face detection, a truth bias has been previously observed^18^ with people demonstrating a tendency to classify faces as real.

The existing research suggests that humans have a limited ability to distinguish real faces and voices from synthetic ones. These previous studies, however, have typically focused on the detection of one modality, employing between-participant designs, and therefore it remains unclear whether synthetic detection ability is consistent within an individual. To this end, we pose the question: **is the ability to distinguish between real and synthetic content a domain-general trait across faces and voices**?

Research adjacent to synthetic content detection has traditionally emphasized modality-specific processing systems, with distinct perceptual mechanisms supporting the identification and recognition of faces and voices^19,20^. There are, however, other findings that suggest face and voice processing do not work completely independently, and instead processing converges onto shared, person-level representations^21^. Empirical evidence for above-chance face and voice identity matching supports the existence of potential cross-modal representations^22,23^, raising the possibility that judgments about identity may draw on modality-general mechanisms rather than being strictly tied to individual sensory modes. Based on these findings, it is plausible that judgments of authenticity and realness are also influenced by modality-general mechanisms.

Whether the human mechanisms for differentiating between real and synthetic content are also modality-specific is yet to be fully established. There is some evidence that this could be a modality-general human skill: for example, the finding that increased face identification performance is linked to increased voice identification performance^24^ indicates that modality-general abilities do exist when working with real faces and voices. Furthermore, research has revealed a correlation between face recognition ability and detection of synthetic faces in a typical population^10^ when presented with faces individually.

We seek to identify a possible domain-general detection ability in typical populations when working with real and synthetic content. If such a domain-general ability exists, then individuals’ performance in detecting synthetic faces and synthetic voices should be positively correlated. The present study directly tests this prediction using a within-participant design, thereby addressing a key gap in the current literature. Therefore, we present our main hypothesis for testing as:

**There exists an ability to detect synthetic stimuli that is independent of stimulus modality**.

It is also beneficial to understand more about the metacognition of individuals, that is how aware are they of their own performance when given no feedback as to their success on the task? This relationship between confidence and accuracy has been previously tested in detection tasks where it was found that people are overconfident in their ability to identify synthetic faces, that is high confidence and lower accuracy^25^, as well as in synthetic videos^26^. It has yet to be tested in a within-participant context to explore whether individuals are confident in detection across different modalities, or whether they exhibit modality-specific confidence. To this end, our second hypothesis is:

**High confidence in an ability to detect AI-generated synthetic faces will correspond with high confidence in ability to detect AI-generated synthetic voices**.

In this study, we expand on the current understanding by empirically testing whether a correlation exists for distinguishing between real and synthetic faces and voices, followed by testing for an effect of reported confidence between modalities.

## Methods

The study received ethical approval from Lancaster University Faculty Science and Technology Research Ethics Committee (ID: FST-2025-5370). All participants gave informed consent before participating in the study, and all methods were performed in accordance with the relevant guidelines and regulations including the declaration of Helsinki.

### Participants

Participants were recruited from Prolific.com, an online participant recruitment platform. The study listing was only shown to participants who met the criteria of having: an approval rating of over 95%, being fluent in English (responding “English” to the Prolific screening question, “Which of the following languages are you fluent in?”), having normal or corrected-to-normal vision (responding “Yes” to “Do you have normal or corrected-to-normal vision?”), no reports of color blindness (responding “No” to “Do you experience colorblindness?”), no self-reported hearing loss or listening difficulties (responding “No” to “Do you have any hearing loss or hearing difficulties?”), as well as to not have a cochlear implant (responding “No” to “Do you have a cochlear implant?”), and to be aged between 18 and 30. The age restriction was set to minimize the likelihood of recruiting participants with cognitive ageing and hearing loss. Data collection occurred over a 17 day period with 13 separate listings (restricted to new respondents only) with a researcher actively monitoring responses.

A sample of 200 was targeted based upon available funds for participant compensation. Of the 200 recruited, 11 participants received payment despite not completing the study due to issues with the hosting server and data were not provided. Of the remaining 189 participants, five (2.50%) self-reported not having paid attention during the study and one participant did not respond to all face trial responses—these six participants were removed. Eleven (5.50%) participants were identified as “monotonic responders”, that is, the face and voice tasks were similar in how participants could respond (e.g., classification, confidence ratings, distinctiveness ratings, attribute usage). Whilst a consistent response within one of these tasks (e.g., classifying all faces as being synthetic, reporting equal confidence in all decisions) was a potentially reasonable and realistic response pattern, when a participant responded identically across the face and voice tasks (e.g., classifying all faces as being synthetic and all voices as being synthetic), this was considered as an unlikely response pattern. The removal of monotonic participants was a deviation from the preregistration, their exclusion did not affect the key confirmatory findings in terms of direction or significance. Following exclusion of these participants, data from 172 participants were used in the following reported analyses.

Demographic information was gathered via Prolific. Of the 172 participants, 75 self-reported as female and 97 as male. The mean age was 24.91 (SD = 3.45). Participant ethnicity is shown in Table 1. Participants were paid £5 for participation, and the average completion time was 29 minutes. This study was pre-registered via the Open Science Framework: https://doi.org/10.17605/OSF.IO/J2MWY.

**Table 1:** Participant demographics.

Ethnicity	Sex	Count
Asian	Female	6
Asian	Male	12
Black	Female	45
Black	Male	46
Mixed	Female	1
Mixed	Male	5
Other	Female	1
Other	Male	3
White	Female	22
White	Male	31

### Materials and methods

#### Faces dataset

Forty unique faces were selected, 20 were real and 20 were synthetic. The real faces were sampled from the Flickr-Faces-HQ Dataset^27^ as used in Nightingale and Farid^7^, and the synthetic faces were generated in Adobe Firefly (a diffusion model) by McGuire et al.^11^. For full details on the dataset curation, please refer to the original articles. Ethnicity and gender were matched between the real and synthetic faces. Face demographics are shown in Table 2.

**Table 2:** Descriptives of the stimuli used in the face classification task.

Ethnicity	Gender	Count	
		Synthetic	Real
Black	Female	6	6
Black	Male	5	5
East Asian	Female	1	1
East Asian	Male	5	5
South Asian	Female	7	7
South Asian	Male	5	5
White	Female	6	6
White	Male	5	5

The face stimuli were split into blocks of eight faces balanced for ethnicity (Black, East Asian, South Asian, White), gender (male or female), and image type (real or synthetic). Participants saw all 40 images with block order and item order randomized to prevent potential ordering effects. The blocks ensured that ethnicity and image type was evenly distributed across the task.

#### Voices dataset

The voice samples used came from Lavan et al.^14^. The human test stimuli were sentences from The Rainbow Passage, selected from 40 speakers in the VCTK database^28^. The same 40 speakers were also cloned using the Instant Voice Cloning tool by ElevenLabs (elevenlabs.io), thus yielding a full stimulus set of 40 real and 40 synthetic voices (for full details on the dataset curation, please refer to the original article). In the present study, the voices were separated into two sets. Each set contained 20 real voices and the voice clones of the other 20 speakers; this ensured no participant would hear a real voice and synthetic voice associated with the same speaker. Participants were randomly assigned to one of these stimuli sets.

#### Procedure

The study was built in PsychoJS and hosted on Pavlovia. Attempts to complete the study from a device other than a laptop or computer (such as a mobile or tablet) were automatically prevented using the experimental software. Having given informed consent, participants were asked to confirm they were using a laptop or computer and that they had normal or normal-corrected vision and hearing. If they answered no, participants were screened out. Participants were told that if they were inactive for more than five minutes during trials, they would be screened out and not receive payment. Participants who failed more than one attention check or were inactive for more than five minutes were screened out and not included in the final dataset.

#### Face task

**Fig. 1:**
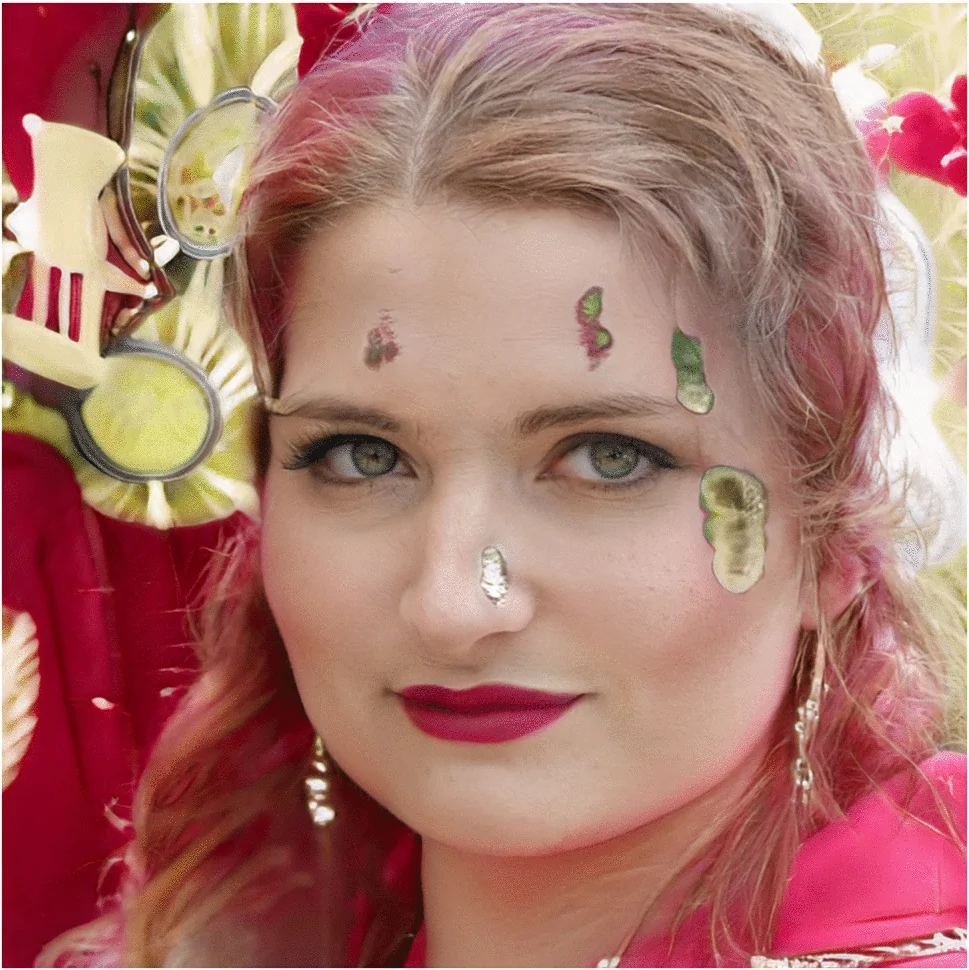
An example of an attention check image shown to participants during the training. Participants received feedback regarding the artefacts in the image that were indicative of AI generation. Image source: Pearson et al.^29^.

The face task began with a brief training session, where participants saw four labelled real faces, four labelled synthetic faces, and four labelled attention check examples. They had unlimited time to familiarize themselves with these examples. The attention check images contained artefacts that resulted in non-realistic looking faces. An example of one such image is shown in Fig. 1.

They then completed three practice trials in which they were asked to classify a real face, a synthetic face, and an attention check image. Participants received feedback in these practice trials but were informed that they would not be given feedback in the main experimental task.

Participants were presented images in a randomized order and classified each face individually. They were shown a centered fixation point for .5s before the face was displayed for 2.75s (the average duration of the voice samples). After presentation, participants were presented with the statement, “This face is:” and were forced to respond either “of a real person” or “artificially synthesized”. They then reported their confidence in their answer (on a five-point numeric Likert scale ranging from 1 “Not at all confident” to 5 “Extremely confident”) and indicated “How much would this face stand out in a crowd?” using a five-point numeric Likert scale ranging from 1 “Not at all” to 5 “Extremely”).

Once all faces had been classified as real or synthetic, participants were asked to report the extent to which they had used image attributes during their decision-making. The attributes included were those identified by Miller et al.^25^. Participants were presented with the question, “To what extent did you use the following attributes to help decide if each of the faces you have just seen were real or artificially synthesized?” with eight options of whether faces were proportional, how alive in the eyes they were, familiarity, memorability, face symmetry, how attractive they were, smoothness of the skin, and the image quality. Responses were on a five-point numeric Likert scale ranging from 1 “Not at all” to 5 “All the time”.

#### Voice task

Before the voice task began, participants were asked to wear earphones/headphones and to “complete the study in one sitting with no distractions”. The voice task followed the same flow as the face task, where participants heard four labelled real, four synthetic voices, and an attention check voice (that instructed participants to “select the response artificially synthesized”) and then completed three practice questions with feedback (one real, one synthetic, one attention check). They were then told they were moving on to the main task and feedback would not be provided. A play button was presented on screen, and clicked the button to listen to the sample before moving on to the questions. The sample could only be played once. They were then asked to classify the voice as real or synthetic, before rating their confidence and distinctiveness of the voice (“how much would this voice stand out in a crowd?”) on the same scales as for the face task.

Following this, participants were asked the question, “To what extent did you use the following attributes to help decide if each of the voices you have just heard were real or artificially synthesized?”. Eight options of: inflection (how the words were spoken with emphasis or emotion), breathing (e.g., breaths between words), background noise (the non-verbal sounds in the clip), pauses (the gaps between spoken words), conversational (how casual the voice sounded), monotone (the absence of any emphasis or emotion), pace of speech (how consistent or rushed the speech was), and microphone quality (e.g., how good the recording sounded) were available. These options corresponded to the top attributes that people have previously reported to using to help classify real and synthetic voices^13^.

Task order was counterbalanced, with half the participants completing the face task first. Once both tasks were completed, participants were asked whether they had fully paid attention during the entire study and assured their answer would not affect payment. A debrief was then presented explaining the study’s aims.

### Analysis

Responses from the face and voice classification task were used to calculate measures of signal detection, specifically d-prime (*d’*) and criterion (*c*). As a measure of sensitivity, *d’* captures the ability to discriminate between noise and signal - that is, an individual’s sensitivity to the information given and how this influences their decision^30^. Positive *d’* values indicate greater sensitivity and represent good performance in a detection task. A value of zero indicates chance performance, and negative values indicate reversed performance (mistaking signal for noise and vice versa). In this study, the signal represents human faces/voices, and noise represents the synthetic stimuli. *c* is a measure of response bias - the necessary information, or threshold, required for an individual to decide that a signal is present. In the present analysis, positive *c* values reflect a conservative response bias which indicates a tendency to respond “synthetic” (more information would be required to agree the signal is present), while negative values reflect a liberal response bias which means participants tend to respond “real” (more willing to agree a signal is present). These values were computed in R using the psycho package^31^. Both *d’* and *c* were calculated for each participant for all trials, as well as for real and synthetic faces separately. Values were computed from counts of *hits* (responding “real” to a human stimulus), *correct rejections* (responding “synthetic” to a synthetic stimulus), *misses* (responding “synthetic” to a human stimulus), and *false alarms* (responding “real” to a synthetic stimulus). From these values, the hit rate and false alarm rate can be derived to calculate *d’*.

Metacognition, the awareness of one’s own thought processes, can be assessed using type II Receiver Operating Curves (ROC). These represent an individual’s performance on a binary classification task whilst accounting for confidence ratings. The area under the ROC (or AUROC) can be used to estimate metacognitive performance. When AUROC is equal to .5, metacognitive performance is at chance^32^ and indicate no insight into their own internal processes. Values closer to 1 indicated good awareness of their own ability, whereas values closer to 0 indicate poor awareness where they are more confident in incorrect responses than correct responses.

## Results

In terms of accuracy, participants correctly classified 65.48% (95% CI = [63.75, 67.21]) of faces which was significantly above chance as assessed by a one-sample t-test, *t*(171) = 17.64, *p* < .001, *D* = 1.35. They correctly classified 53.95% (95% CI = [52.64, 55.27]) of voices, which was also significantly more than chance, *t*(171)= 5.94, *p* < .001, *D* = .45.

**Fig. 2:**
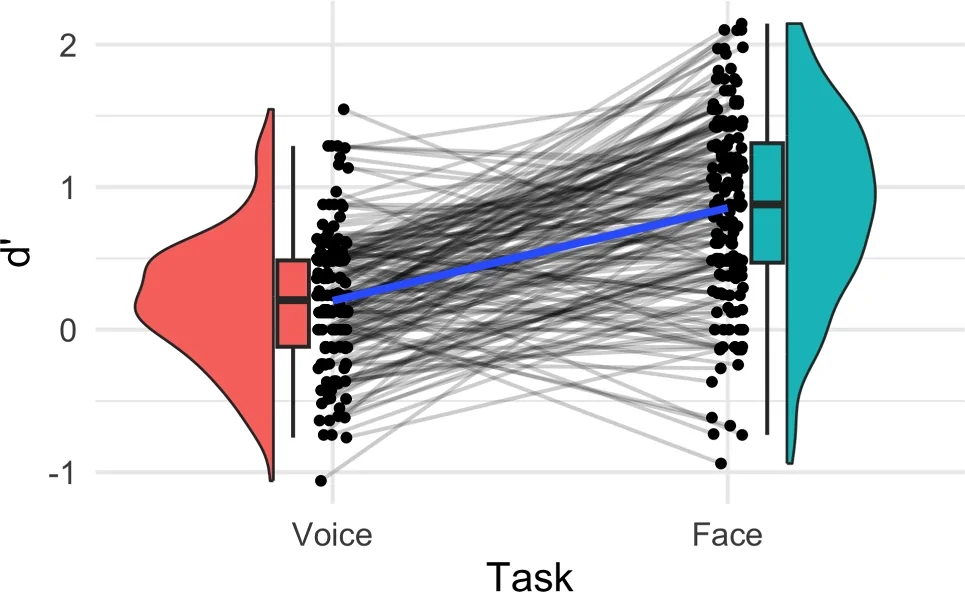
The black dots represent individual data points to show each participant’s performance on the face and voice detection tasks. The boxplots show the median *d’* and distribution. The lines between the black dots on the left and right show overlaid pairing indicating individual trends. The blue line shows the average trend and distribution of *d’* values on the two detection tasks.

A paired-samples t-test conducted on *d’* values indicated that on average, participants performed significantly better at discriminating between real and synthetic faces (*d’* *M* = .85, 95% CI = [.76, .95], SD = .64) relative to discriminating between real and synthetic voices (*d’* *M* = .20, 95% CI = [.13, .27], SD = .45), *t*(171) = 11.76, *p* < .001, indicating a large effect, *D* = .90. This difference is presented in Fig. 2.

### Objective stimuli similarity

The similarity of the faces was determined via the pre-trained face recognition model ArcFace^33^. The model computed cosine similarity, ranging from -1 (most dissimilar) to 1 (identical/most similar) between the extracted face features of all faces as carried out in^9^. A mean similarity score was computed for each face against all other faces, as well as similarity to the nearest neighbour of each face in a multidimensional space. Voice similarity was computed using the pre-trained voice recognition model SpeechBrain ECAPA-TDNN^34^. Cosine similarity between all audio samples were calculated, before being subset into the two sets (A and B) to calculate similarity and nearest-neighbour scores. Subsetting was used to avoid similarity scores being influenced by the relationship between the real and cloned speaker that were intentionally placed in separate sets.

To characterise the objective properties of the stimulus set, we quantified inter-item similarity and stimulus distinctiveness separately for face and voice stimuli using deep embedding models. For each stimulus, we computed three metrics: mean similarity to all other stimuli within the same modality, nearest-neighbour similarity to stimuli from the opposite condition (real or synthetic), and the margin between nearest same-condition and nearest cross-condition neighbours, indexing how cleanly each stimulus is separated from the opposing category in embedding space. Face and voice stimuli differed significantly on all three metrics. Voice stimuli had significantly higher mean inter-item similarity than face stimuli, *t*(117.92) = −18.87, *p* < .001, as well as higher cross-condition nearest-neighbour similarity, *t*(111.02) = −17.84, *p* < .001, indicating that the voice embedding space was more compressed overall. Face stimuli showed a significantly larger mean margin, *t*(88.37) = 3.36, *p* = .003, suggesting that individual face stimuli were more objectively distinctive (between real and synthetic) than voice stimuli. All p-values were Bonferroni-corrected for three comparisons.

**Fig. 3:**
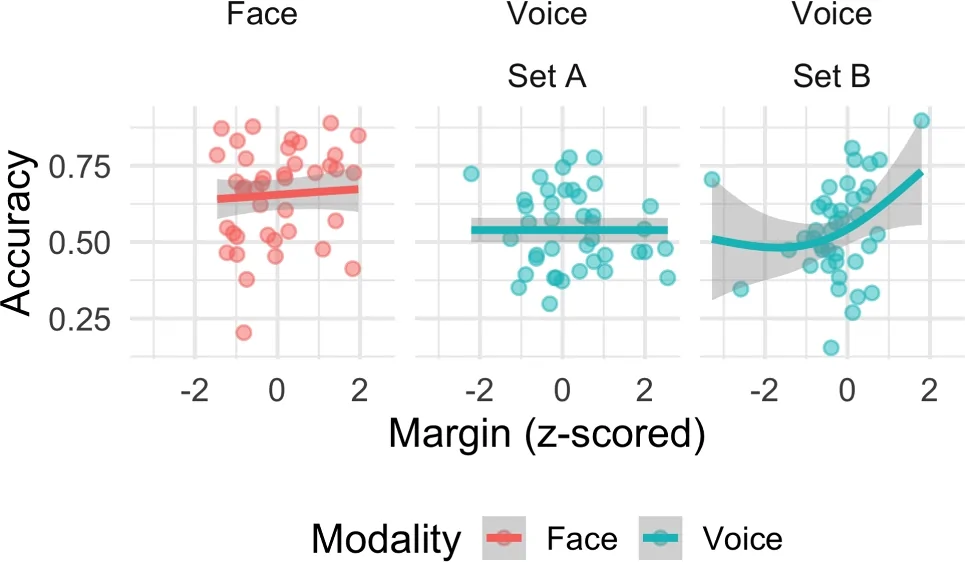
Classification accuracy against z-scored margin between nearest same-condition and nearest cross-condition neighbours, separated by modality and set type (for voices).

To assess whether objective stimulus properties accounted for differences in identification accuracy across modality, the z-scored margin values (standardised within modality to account for differences in embedded space), along with the modality and their interaction were tested. No significant contribution was seen for either margin *β* = 0.031, *t* = 1.31, *p* = .19) or its interaction with modality *β* = −0.024, *t* = −0.81, *p* = .42). As reported above with *d’* between modality, modality was a significant predictor *β* = −0.115, *t* = −3.99, *p* < .001). Despite differences in the distinctiveness of the stimuli across their respective embedding spaces, the higher accuracy for faces was not statistically-attributable to these differences in objective stimulus distinctiveness, with the differences shown in Fig. 3.

### Hypothesis one: there exists an ability to detect synthetic stimuli that is independent of stimulus modality

**Fig. 4:**
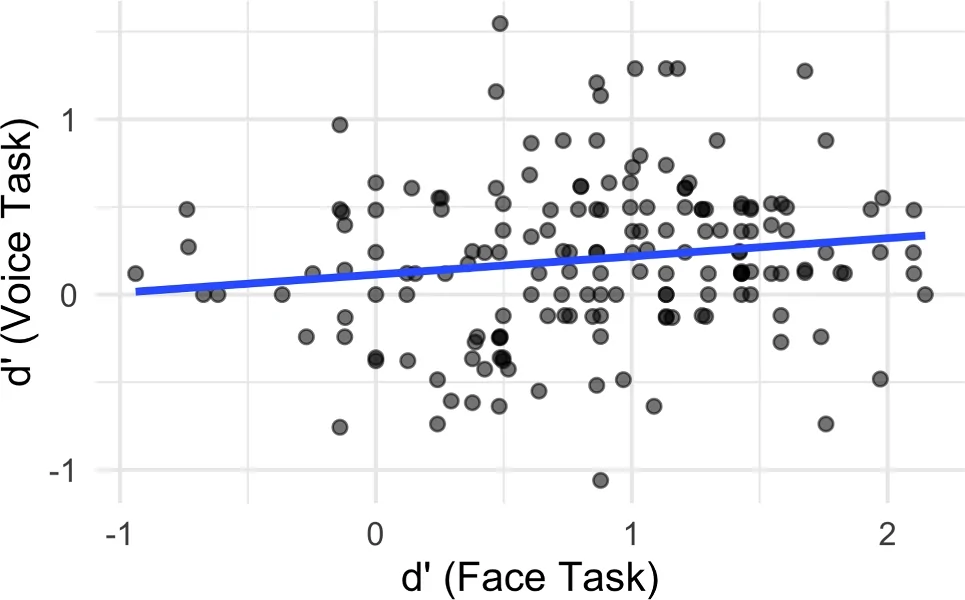
Relationship between *d’* values from the face and voice task. Each black dot represents an individual participant’s scores. Darker points indicate participant overlap.

A simple linear regression was conducted between *d’* values for the face and voice task. This analysis found no significant relationship between performance on the two tasks when regressing voice performance on face performance, *F*(1, 170) = 3.80, *β* = .21, *p* = .053, as indicated in Fig. 4. An R-squared value of .02 is sufficiently small enough that any significant effect potentially identified by an increased sample size would carry very little real-world impact. A power analysis indicates that a minimum sample size of 387 would be required to yield a significant effect of this size, further emphasizing its small influence.

For *c* values, a one-sample t-test was used to assess whether responses to the face and voice tasks were significantly different to a neutral response of zero. Participant responses to faces were significantly more liberal than a neutral approach (*M* = −.24, 95% CI = [−.31, −.18]), *t*(171) = −7.92, *p* < .001, *D* = −.60, and responses to voices had a smaller effect, but were also significantly more liberal than neutral (*M* = −.07, 95% CI = [−.12, −.02]), *t*(171) = −2.85, *p* = .005, *D* = −.22. Thus, for both faces and voices, participants were more likely to respond “human”.

A paired-samples t-test between *c* values from the face and voice task highlighted a significant difference between response strategies, *t*(171) = −4.88, *p* < .001, *D* = −.37 with an average difference of −.18 between the tasks. This suggests participants were more likely to respond “real” towards faces than to voices, albeit being a small-medium effect.

**Fig. 5:**
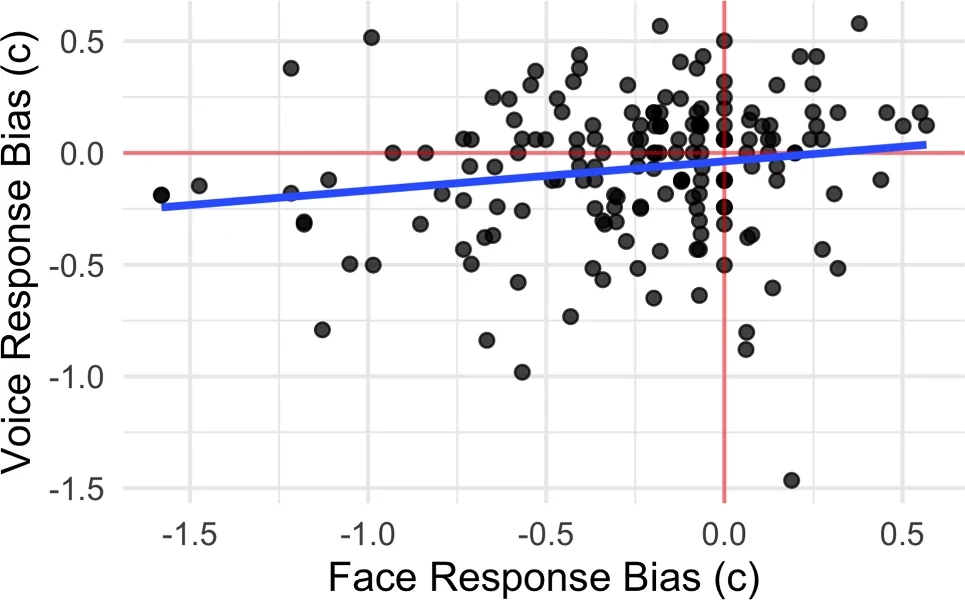
Scatter graph representing the relationship between response bias (c) on the two tasks. For faces, black dots to the left of the vertical red line represent liberal responding (responding “human”) and black dots to the right represent conservative responding (responding “synthetic”). For voices, black dots below the horizontal red line are liberal and above the line are conservative. Darker points indicate participant overlap.

Further, a linear regression was used to assess the relationship between participants’ *c* values on the face and voice tasks. A positive relationship was detected indicating that participants who required higher thresholds for classifying faces as real also required higher thresholds for voices, with *β* = .21, *F*(1,170) = 4.81, *p* = .030, R-squared = .02. The model explains minimal variance, indicating the relationship, whilst significant, is very weak. The relationship between the two tasks and response strategies is shown in Fig. 5.

### Hypothesis two: high confidence in an ability to detect AI-generated synthetic faces will correspond with high confidence in detecting AI-generated synthetic voices

The mean average confidence rating for real faces was 3.75 (SD = .98) and synthetic faces were marginally lower (*M* = 3.73, SD = .99). This trend was reversed for voices, with less confidence in classifying real voices (*M* = 3.79, SD = 1.00) than synthetic voices (*M* = 3.80, SD = 1.00), but with slightly higher confidence for voices than faces. A simple linear regression was used to compare the average confidence ratings participants gave for faces (*M* = 3.74, 95% CI = [3.66, 3.82]) and voices (*M* = 3.79, 95% CI = [3.71, 3.88]). A strong, significant relationship was seen, *F*(1, 170) = 334.44, *p* < .001, R-squared = .66, suggesting that participants responded similarly in terms of their confidence across both tasks.

To assess whether individuals report greater uncertainty when classifying real or synthetic stimuli, confidence ratings were compared for real and synthetic stimuli within modality. No significant effect was seen for faces, *t*(171) = −.77, *p* = .445, indicating that individuals report similar levels of confidence to real and synthetic faces. A similar finding is reported for voices, *t*(171) = .17, *p* = .868.

**Fig. 6:**
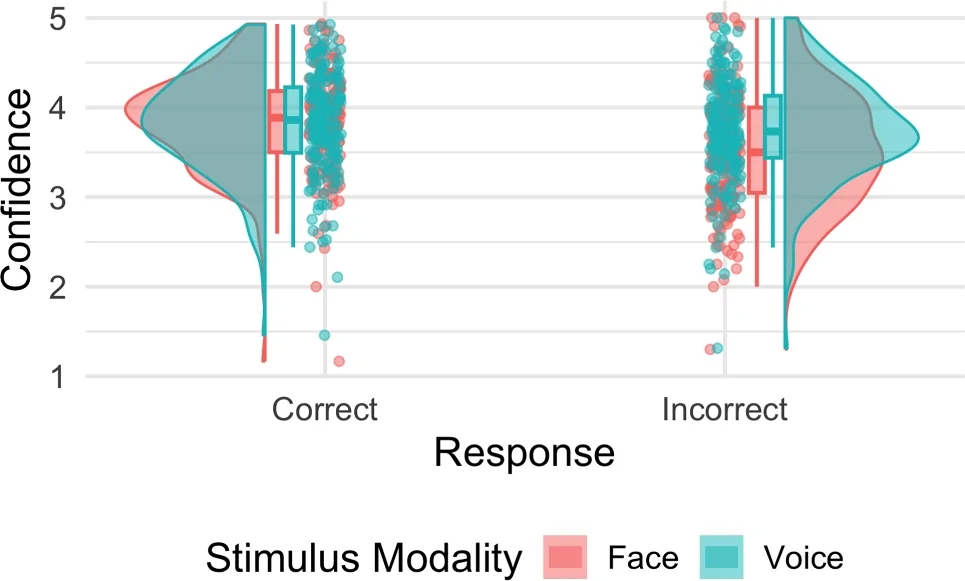
Raincloud plot showing confidence ratings between correct and incorrect responses for both the face and voice task. Each dot shows the mean confidence rating for a participant on each task for correct and incorrect responses. The boxplots represent the median and interquartile range (IQR), and density plots further highlight the distributions.

When separating confidence ratings by whether the response was correct or incorrect, a pattern emerges across both modalities that greater uncertainty exists for incorrect responses. For faces (*M*$_{correct}$ = 3.83, 95% CI = [3.75, 3.91], *M*$_{incorrect}$ = 3.51, 95% CI = 3.41, 3.61]), a paired t-test indicated that confidence ratings for incorrect responses were, on average, significantly lower than confidence ratings for correct responses, *t*(171) = 10.34, *p* < .001, *D* = .79). A smaller, yet still significant, effect was also found for voices (*M*$_{correct}$ = 3.83, 95% CI = [3.74, 3.91], *M*$_{incorrect}$ = 3.74, 95% CI = [3.66, 3.83]), *t*(171) = 3.59, *p* < .001, *D* = .27). The differences are represented in Fig. 6.

**Fig. 7:**
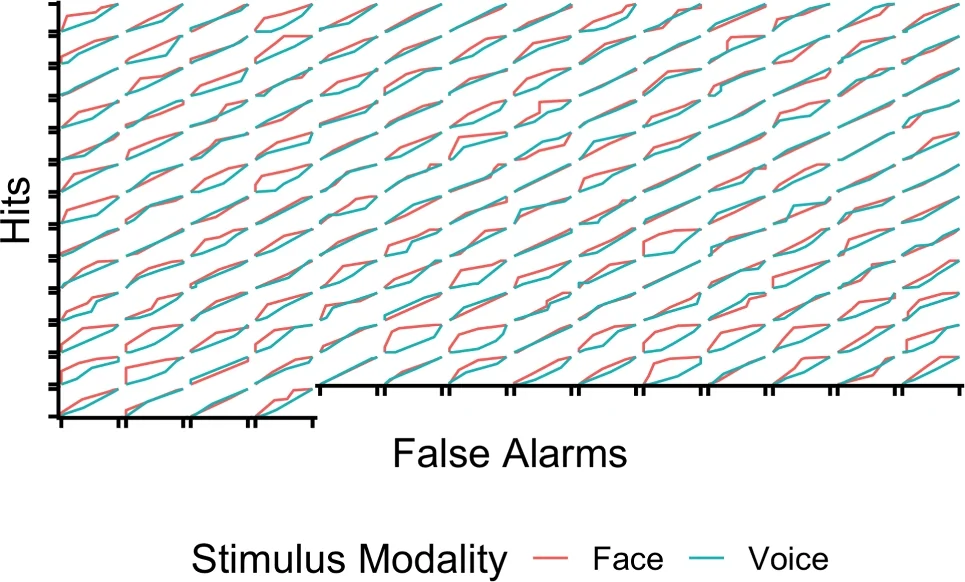
AUROC for both voice and face tasks represented for each participant. Each plot represents an individual with their ROC curves plotted for both face and voice tasks. Straight diagonal lines indicate chance performance and curved lines with apexes towards the top left indicate better-than-chance performance.

In a metacognitive awareness analysis, the AUROC values between the two tasks were compared in a one-sided t-test against a chance-insight value of .5. The AUROC value accounts for confidence ratings against performance with great values indicating greater awareness of their own performance (high ratings of confidence for correct responses and low ratings for incorrect responses). Perfect insight corresponds to a value of 1, chance at .5, and inverse insight (high confidence when incorrect and low confidence when correct) at 0. Participants demonstrated a significant improvement over chance insight for faces (mean AUROC = .59, 95% CI = [.57, .60]), *t*(171) = 10.65, *p* < .001, *D* = .81). For voices (mean AUROC = .48, 95% CI = [.47, .49]), participants performed worse than chance, indicating worse insight than for faces, *t*(171) = −3.42, *p* < .001, *D* = −.26). These findings indicate that participants possess a greater-than-chance insight into their performance on the face task but display poor insight into their performance on the voice task, often reporting greater confidence in incorrect responses than correct ones, as displayed in Fig. 7.

### Exploratory analyses

In this section, we report on the preregistered exploratory analyses that were unlinked to hypotheses.

#### Hyperrealism and averageness

Other researchers have observed a hyperrealism effect where (GAN-generated) synthetic faces were more frequently reported as real relative to human faces^25^. This effect is not detected in the present dataset for faces, where real faces were classified as real (*M* = 36.82%, 95% CI = [35.62, 38.01]) significantly more frequently than synthetic faces (*M* = 21.34%, 95% CI = [19.87, 22.80]), *t*(171) = 17.64, *p* < .001, *D* = 1.35. The same holds for real voices (*M* = 28.24%, 95% CI = [27.12, 29.36]) compared to synthetic voices (*M* = 24.29%, 95% CI = [23.17, 25.41]), *t*(171) = 5.94, *p* < .001, *D* = .45.

Real faces were considered to be less distinct (more average) than synthetic faces. A paired samples t-test reported a significant mean difference of −.13 between real (*M* = 3.05, 95% CI = [2.95, 3.16]) and synthetic faces (*M* = 3.18, 95% CI = [3.09, 3.28]), *t*(171) = −4.94, *p* < .001, *D* = −.38). This finding contrasts with previous findings that used GAN-generated faces rather than Diffusion who reported that synthetic faces have passed through the “uncanny valley” and are indistinguishable from real faces^7,25^.

This difference in distinctiveness was not seen for voices, with a paired t-test finding no significant difference between real (*M* = 3.21, 95% CI = [3.10, 3.31]) and synthetic (*M* = 3.22, 95% CI = [3.11, 3.32]), *t*(171) = −.47, *p* = .639. This suggests that, within the bounds of the experimental design, real and synthetic voices were considered non-significantly different in terms of distinctiveness.

#### Attribute use

**Table 3:** Descriptive statistics and binomial mixed-effect regression model results for the associations between participants’ reported face attribute use and (1) judgments of a face as real and (2) correct classification of a face as real or synthetic. Values in bold are significant at the .05 threshold.

Attribute	Descriptives		Real		Accuracy	
	Mean	SD	Beta	*p*	Beta	*p*
Proportional	3.46	1.16	.04	.423	−.04	.357
Alive in the eyes	3.98	1.05	−.01	.838	−.01	.825
Familiar	2.84	1.26	.03	.540	<.01	1.000
Memorable	2.77	1.17	−.01	.892	−.04	.299
Symmetrical	3.68	1.02	−.01	.773	<.01	.943
Attractive	2.89	1.30	.06	.162	<.01	.943
Smooth skinned	4.10	1.07	**−.12**	**.015**	**.12**	**.004**
Image quality	4.40	0.79	−.07	.303	.07	.239

To assess attribute use, two models were used for both the face and voice modalities; one model tested attributes against judging a face/voice as human (i.e., what attributes do people *think* makes a stimulus real), and another model tested the attributes against correct classifications (i.e., what attributes are used in correctly identifying real and synthetic stimuli). Binomial mixed-effect regression models were used on the item-level with a random effect of participant.

Of the eight attributes for faces, skin smoothness was a significant factor associated with judging faces as synthetic as opposed to real, *β* = −.12, *p* = .015, and in terms of accuracy, skin smoothness was also a significant attribute used as a positive predictor of performance, *β* = .12, *p* = .004. That is, participants reported using skin smoothness less when they judged a face to be real (regardless of accuracy), but those who reported an increased use of skin smoothness to make decisions did achieve greater accuracy. All attribute values are reported in Table 3.

In judging a voice as real, inflection was a significant positive predictor, *β* = .11, *p* = .027, however when making correct choices, inflection was no longer a significant predictor and instead, breathing (such as the breathe between words) was a significant attribute for positively predicting accurate responses, *β* = .06, *p* = .016. All attribute values are reported in Table 4. This can be understood as that people used inflection to decide if a voice was real, but it did not correspond to accuracy, whereas a focus on breathing was more likely to correlate with higher accuracy levels.

**Table 4:** Descriptive statistics and binomial mixed-effect regression model results for the associations between participants’ reported voice attribute use and (1) judgments of a voice as real and (2) correct classification of a voice as real or synthetic. Values in bold are significant at the .05 threshold.

Attribute	Descriptives		Real		Accuracy	
	Mean	SD	Beta	*p*	Beta	*p*
Inflection	4.13	0.82	**.11**	**.027**	.04	.236
Breath	3.80	1.22	-.04	.262	**.06**	**.016**
Background noise	3.02	1.26	.01	.814	−.04	.117
Pauses	3.79	1.06	−.01	.741	.01	.860
Conversational	4.08	0.94	−.04	.362	−.04	.205
Monotone	3.99	1.00	−.06	.170	−.02	.505
Pace	4.15	0.96	<.01	.956	−.04	.182
Mic quality	3.75	1.22	.04	.195	<.01	.934

## Discussion

The current study is, to our knowledge, the first to employ a within-participants design to examine people’s ability to distinguish between real and AI-generated stimuli across two modalities: faces and voices. For Hypothesis One, we found no evidence of a significant relationship between face and voice classification performance, indicating the possibility that (at least for the specific face and voice stimuli chosen) a modality-general realness detection ability does not exist. Supporting Hypothesis Two, participants responded similarly in terms of their confidence in their responses across modalities, regardless of actual performance. In exploratory analyses, greater uncertainty was reported for incorrect responses than for correct responses, suggesting some insight into one’s own performance—this insight was more pronounced for faces than for voices. When examining how participants’ reported use of stimuli attributes affected performance, we found only one attribute to be diagnostic for each task: skin smoothness for face task performance and breathing for voice task performance. Our results suggest an absence of a modality-general realness detection ability for static face and voice clone stimuli. The findings also indicate that insight into task performance is not consistent across the two modalities, with face task performance being more closely linked with confidence.

The core finding—that no modality-general effect was detected—contrasts with the expected outcome for Hypothesis One. Specifically, we found no evidence for an underlying modality-general synthetic detection ability across static face images and voice audio. Although this null effect may partially reflect experimental design choices, it also provides initial evidence against the existence of a unified, modality-general “realness detection” mechanism. Instead, synthetic content detection may be better characterised as relying on modality-specific processes.

Prior work offers grounds for expecting cross-modal links in this domain. Moderate correlations between face and voice recognition performance have been reported, particularly among Super-Recognizers^24^, and face recognition ability has been linked to synthetic face detection in typical observers^10^. These findings suggest a potential shared component underpinning both identity processing and synthetic content detection. However, this relationship appears fragile: it does not consistently extend to more complex or dynamic stimuli such as video^35^, and face recognition ability itself shows substantial inter-individual variability in processing strategies and capacities rather than reflecting a single shared mechanism^36^.

A more fundamental explanation may lie in a mismatch between the task demands of identity recognition and synthetic content detection. Identity recognition depends on forming and maintaining stable representations across repeated exposures^19,37^, whereas synthetic content detection may instead be driven by sensitivity to statistical irregularities, artefacts, or deviations from learned distributions^7,38^. These tasks likely draw on partially distinct processes, which helps explain not only the present null result but also inconsistencies across the broader literature. Dunn et al.^10^ and Ramon et al.^35^, for instance, both examined Super-Recognizers yet reported contrasting findings depending on stimulus type—suggesting that observed effects are contingent on stimulus properties rather than reflecting stable, generalizable, human abilities. Beyond task structure, perceived realness is also shaped by contextual factors. Lavan et al.^14^ demonstrated that real voices were judged as less “real” when presented alongside high-quality voice clones, than when real voices were compared to more artificial text-to-speech systems indicating that realness judgments are context-dependent. Taken together, realness perception may not be governed by a fixed, modality-general mechanism, but instead emerges from an interaction between observer-specific perceptual abilities, stimulus, and broader context.

An alternative explanation for the absence of a modality-general effect is that advances in generative models have reduced the perceptual gap between real and synthetic stimuli to the point of practical indistinguishability. Under this account, human observers may increasingly default to classifying stimuli as real, reflecting a shift in decision criterion rather than sensitivity per se. If so, pure human detection may become unreliable, necessitating the integration of algorithmic support. Early evidence suggests that human performance can be improved when offered algorithmic feedback^39^, although this introduces new risks, such as over-reliance on algorithmic judgments^29^.

Across both modalities, participants showed a consistent tendency to respond “real” rather than “synthetic”. This bias was stronger for faces (*D* = −.60) than for voices (*D* = −.22), aligning with prior findings in both domains^13,14,25^. One interpretation is that despite explicit awareness that stimuli may be synthetic, participants may be predisposed to treat socially meaningful signals—such as faces and voices—as genuine^40^. This pattern is consistent with a broader “truth bias” or “truth-default” whereby individuals will default to assuming authenticity unless sufficient evidence prompts suspicion^17,18^.

In the present study—as in most synthetic content detection paradigms—participants were explicitly informed that stimuli could be either real or synthetic. This instruction should have provided a reasonable trigger for suspicion, inducing a more analytical approach than would occur in naturalistic settings. Consequently, the observed response bias may in fact *underestimate* the extent to which individuals assume stimuli to be real outside the experimental setting. This highlights a broader limitation of the literature: performance in explicitly framed detection tasks may not generalize well to real-world contexts, where suspicion is not necessarily primed and detection rates may be substantially lower than observed.

Methodological factors relating to stimulus presentation further qualify the interpretation of performance. Here, exposure was constrained: voice samples could be played only once, and face stimuli were presented for 2.75 seconds. This design intentionally removed the opportunity for extended scrutiny or repeated inspection. By contrast, previous studies have often allowed unlimited viewing time for faces (e.g.,^7,11,25^) or repeated listening for voices (e.g.,^41^). Despite these constraints, participants in the present study still performed above chance for both modalities. This suggests that extended exposure may not substantially enhance detection performance. More importantly, it raises the possibility that synthetic content detection relies primarily on rapid, perceptual processes rather than deliberate, effortful scrutiny. At the same time, with the explicit task priming, if participants are already operating in a heightened analytical mode, any additional benefit of increased exposure time may be attenuated.

Finally, the similarity structure of the stimulus set did not predict classification performance for either faces or voices. This result aligns with recent evidence showing that statistical averageness—a proxy for typicality—does not reliably predict the perceived realism of GAN- or diffusion-generated faces^38^. Together, these findings suggest that perceived realness is not simply a function of how typical or “average” a stimulus appears relative to others, but instead depends on more complex and potentially higher-order perceptual or contextual cues.

### Confidence and metacognitive awareness

Dunn et al.^10^ reported that individuals with above-average face recognition ability exhibit greater confidence in correct than incorrect decisions, indicating preserved metacognitive sensitivity. The present findings replicated this pattern in a broader population: for faces, this related to an effect size of .79, and an effect of .27 for voices. Taking this further and integrating confidence ratings into an insight analysis (AUROC), the face insight was preserved, however it was not seen for voices, with participants demonstrating worse-than-chance insight. This suggests, that specifically for voices, confidence ratings should be treated cautiously, particularly given that overall voice classification accuracy was itself only marginally above chance level at 53.95%.

This asymmetry is consistent with established modality differences in person perception. Humans are typically more accurate at recognising faces than voices^42^, more confident in face-based judgments^43^, and more robust to distraction during face processing^44^. Extending this to synthetic content, participants were not only more accurate when classifying faces than voices, but also better calibrated in their confidence for faces, suggesting a more stable and reliable perceptual basis for judgment in this modality.

#### Hyperrealism and attributes

Miller et al.^25^ reported a hyperrealism effect for faces, whereby synthetic faces were more likely to be judged as “real” than genuine human faces. This effect was attributed to increased averageness in synthetic faces, consistent with face-space accounts in which typical faces are represented closer to a central prototype and are therefore perceived as more veridical^45,46^. However, more recent evidence suggests that proximity to the face-space mean does not reliably predict perceived realism in synthetic faces^38^, calling into question whether averageness alone can explain hyperrealism effects.

In the present study, real faces were more likely to be judged as “real” than synthetic faces, and were also rated as less distinctive. This opposes the “hyperrealism” effect^25^. Several methodological differences may account for this divergence. First, we used diffusion-generated images^11^ rather than StyleGAN2-generated faces^7^. Diffusion models may simply produce faces that are either less “hyperreal” or, conversely, more varied and distinctive, positioning them further from the central face-space prototype. Second, our stimulus set included racial diversity (Black, East Asian, South Asian, and White faces), whereas Miller et al. examined only White faces within a Caucasian American sample. This raises the possibility that hyperrealism is not a general property of synthetic faces, but instead depends on the interaction between stimulus characteristics and observer experience. Given well-established own-race biases in face recognition^47^, it is plausible that hyperrealism effects are similarly constrained, emerging only when synthetic faces align closely with the observer’s internalised face-space. Under this account, hyperrealism would not reflect a universal perceptual bias toward averageness, but rather a context-dependent effect shaped by the correspondence between stimulus distributions and observer-specific experience. A parallel pattern was observed for voices: Consistent with the face results, and in line with previous research using the voice dataset, we did not observe hyperrealism in voice perception^14^. Real and synthetic voices were rated as equivalent in terms of averageness, which may account for the absence of any bias toward synthetic stimuli.

When deciding whether a face was real or artificially synthesized, participants who were more likely to classify faces as synthetic reported skin smoothness as a significant attribute for decision-making, replicating previous findings^25^. The present study also found skin smoothness was used as a reliable diagnostic cue, with increased detection rates linked to higher focus on skin smoothness. Skin smoothness may be regarded as a feature of synthetic content as models are trained on vast numbers of faces in which variations that would otherwise disrupt skin texture are effectively averaged out.

For voices, inflection (“how the words were spoken with emphasis or emotion”) was the only significant attribute used to determine whether a voice was human. Inflection, and other linguistic features, have been previously reported as being the main factors used for decision making^13,48^, suggesting that people focus on the rise and fall in pitch over other features in the speech. One potential reason for this could be that people hold expectations for how synthetic voices should sound, perhaps driven by frequent exposure to Text-To-Speech voices that typically lack humanlike-intonation^48^. For example, many people expect that the presence of an accent is a strong indicator for a real person based on a presumption that AI-generative models cannot generate convincing accents^49^.

### Implications for the real world

Beyond a theoretical understanding of how people classify synthetic stimuli, it is also important to consider the potential practical implications, while taking care not to overinterpret a null finding. If no true modality-general realness detection ability exists, those working with synthetic content, such as in law enforcement or forensic settings, should not assume skill transfer between modalities. Evidence suggests human detection of synthetic content is at or near chance across modalities^8^, implying professionals may need to specialize by medium (e.g., image, video, audio). Similarly, expert testimony in legal contexts should be tied to the specific modality of demonstrated expertise; conflating skills across modalities risks undermining credibility and integrity. The current findings show that greater confidence was expressed for correct classifications than incorrect classifications for both faces (*D* = .79) and voices (*D* = .27). In addition, the metacognitive analysis revealed an above-chance metacognitive insight for face classifications and below-chance metacognitive insight for voices. Taken together, these results suggest that individuals have limited insight into their ability to classify faces and extremely limited insight when classifying voices. As the study did not include Super-Recognizers, it remains possible that performance insight may be higher in these populations.

### Limitations and future work

Prior work suggests that performance depends on both the stimuli and task design^50^. One limitation of this study was that task difficulty was not standardized across modalities, meaning one task may have been inherently harder than the other. The voice task, which produced near-chance accuracy (with 35.47% scoring at or below chance compared to 12.80% for faces), may have been particularly difficult. Despite this, some participants performed well above chance, suggesting this was not a universal floor effect. To reduce disparities between stimulus types, both tasks were presented once and matched in presentation duration. Lowering the difficulty of the voice task to match that of the face task would have not only have undermined the study’s ecological validity, but would also have relied on the unfounded assumption that synthetic face and voice detection are comparable abilities. It is possible that synthetic faces are simply easier to distinguish from real faces than synthetic voices are from real voices. Even with differing task difficulties, any modality-general detection ability should still manifest through correlated performance. Future research should clarify the mechanisms driving modality-specific detection. Training interventions, such as highlighting artefacts or providing examples, have shown little improvement in detection accuracy for faces or voices^12,26,41^, as synthetic content becomes more sophisticated, effective human-based interventions may remain limited. Identifying high-performing individuals and the traits that support their success could help guide the development of more targeted strategies for improving synthetic content detection.

## Conclusion

Human decision-making remains a significant part of the process of classifying real and synthetic content, specifically when handling faces and voices. Although between-participant effects have been tested in both domains, the within-participant relationship has, up to now, been unreported. In a study that tested the ability to distinguish between real and synthetic faces and voices, it was seen that the expected modality-general effect did not arise, indicating the absence of a domain-general mechanism for detecting real content. The findings may be particularly relevant within forensic and legal contexts, where expertise in synthetic content detection should be considered domain-specific (particularly non-transferable between faces and voices), and that insight into performance is limited and not consistent between modalities.

## Data Availability

This paper is a reproducible manuscript with embedded analyses written in Quarto. All necessary data and files required to reproduce all reported findings can be found at: https://doi.org/10.17605/OSF.IO/8JZ6H.
